# Utilization of Prepared Nanocellulose as a Biopolymer for Adsorption Kinetics of Cobalt Ions from Wastewater

**DOI:** 10.3390/polym15092143

**Published:** 2023-04-30

**Authors:** Khaled Bin Bandar, Saad Aljlil

**Affiliations:** Water Management and Treatment Technologies Institute, King Abdulaziz City for Science and Technology, Riyadh 12354, Saudi Arabia; kbandar@kacst.edu.sa

**Keywords:** adsorption, cobalt ions, wastewater, batch adsorber, nanocellulose

## Abstract

This study evaluated nanocellulose derived from discarded palm leaves for its ability to remove cobalt ions from industrial wastewater. The process involved extracting cellulose nanocrystals (CNCs) from date palm leaves through a series of repeated chemical treatments. The study examined the adsorption rates of cobalt ions under various conditions using different techniques. Two equilibrium models, the Langmuir and Freundlich models, were employed, and the Langmuir model was found to be consistent with the experimental data. The maximum amount of cobalt ions adsorbed at room temperature was 5.98 mg/g. Furthermore, several kinetic models were used to gain insight into the adsorption mechanism, including the pseudo-first-order, pseudo-second-order, Elovich, and intraparticle diffusion models. The pseudo-second-order model provided an accurate description of the adsorption process, while the Elovich equation suggested a chemical reaction between cobalt ions and nanocellulose, involving multiple chemical reactions and mass-transfer processes. Kinetic parameters were critical in interpreting the results, and the study’s findings were in agreement with the pseudo-second-order and intraparticle models, indicating general chemical reactions and diffusion resistance.

## 1. Introduction

The use of heavy metals and their compounds in various industries such as printing, electroplating, metallurgy, textiles, metal processing, and ceramics has led to significant concerns about heavy-metal pollution in wastewater in recent years [1]. Cobalt ions (Co^2+^) are one of the most commonly found heavy-metal pollutants in industrial wastewater, and their elimination is vital for preserving a healthy ecosystem. Adsorption, which involves using nanocellulose as a biopolymer adsorbent, has proven to be a promising technique for removing cobalt among the many methods available [1].

Cellulose-based materials are biodegradable and nontoxic, making them ideal candidates for adsorption-based wastewater treatment. The large number of hydroxyl groups on the surface of cellulose improves the adsorption capacity. Nanocellulose and functionalized nanocellulose can significantly improve the adsorption of heavy metals and dyes in wastewater, making them promising materials for future water treatment [2].

Due to their surface properties, nanocellulose materials offer an environmentally beneficial and cost-effective method for absorbing pollutants. This study concentrates on the structural design of water-treatment nanocellulose materials that can target specific contaminants. For instance, a dendrimer poly(amidoamine)-grafted cellulose nanofibril aerogel has a record-breaking Cr(VI) removal capacity. In addition, a cellulose hydrogel-coated mesh with superhydrophilicity and underwater superoleophobicity is effective at separating insoluble oils and is recyclable. These results demonstrate nanocellulose’s potential for future wastewater treatment [3].

This study investigated the absorption efficacy of hydrogels produced by a copolymerization reaction involving nanofibrillated cellulose (CNF), acrylic acid (AA), acrylamide (AM), and N, N-methylene-bisacrylamide (MBA) as a cross linker. According to thermodynamic and kinetic studies, the disruption of the homolytic covalent bonds C6-OH and C3-OH can form cellulose radicals [4].

The crystallinity of rice husks made from highland rice and upland rice types was examined. The research used the highest silica content amorphous ash, metakaolin, aggregates, water, and alkaline activators to create geopolymer mortar prisms. The mortar’s compressive and flexural strengths were tested after seven days and were found to be 1.5 and 1.3 MPa, respectively [5].

Biochar derived from Matamba shells possesses exceptional properties in terms of surface structure, chemical composition, and dynamic behavior. Its asymmetrical honeycomb structure and high carbon, nitrogen, and oxygen content facilitate its efficient adsorption of iodine via attractive forces and stacking interactions. The adsorption kinetics of biochar can be effectively analyzed using the Elovich and intraparticle diffusion models, which accurately describe its properties. With an aromatic ring structure and a carbon content exceeding 50%, biochar has a significant potential to eliminate water pollutants and enhance waste management, particularly in developing nations. Moreover, biochar can contribute to reducing greenhouse-gas emissions and addressing the issue of global warming [6].

Understanding the cobalt ions adsorption kinetics of nanocellulose in a batch adsorber is crucial to determine the process’s kinetic parameters. Therefore, the adsorption of metal ions on the adsorbent must be studied sequentially when describing a batch adsorption process:

1. The metal diffuses from the solution onto the outer surface of the sorbent particle;
2. The metal diffuses through the solution into the pores of the adsorption sites;
3. Metal ions bond with sorbent particles in a chemical reaction.

One or both of the above steps can control the adsorption rate. However, kinetic models usually include only some of the three steps, the rates of which are generally governed by diffusion mechanisms or chemical reaction steps, e.g., a chemical reaction step may be the controlling mechanism.

Thus, studying chemical reaction-based and diffusion-based kinetic models is crucial, as they help understand the rate-limiting mechanism involved.

Researchers have employed chemical reaction models to gain insights into the mass transfer controlling step of metal ions adsorption on various adsorbents [7,8,9,10,11,12,13,14,15,16,17]. Among the extensively studied adsorbents is zirconium oxide (ZrO_2_), which is highly effective in adsorbing Co^2+^ ions, making it a promising candidate for industrial wastewater treatment [7]. Moreover, the pseudo-second-order kinetics demonstrated by ZrO_2_ for Co^2+^ adsorption further validates its high efficiency. Therefore, understanding the mass transfer controlling step of metal ion adsorption on adsorbents such as ZrO_2_ can help optimize the design of effective and economical industrial wastewater treatment systems [7].

The adsorption process of cobalt on silica gel rice husk ash was studied by kinetics. The second-order pseudo model employed in the experiment does not include intraparticle diffusion as a significant influence on the equation [8]. Calcium phosphate precipitated with iron oxide formed magnetite-hydroxyapatite nanocomposites and was used to adsorb cobalt ions. The process is studied by analyzing the effect of stirring speed and temperature changes. Through the kinetic model, it was found that the Co^2+^ adsorption rate followed a pseudo-second-order mode [9]. Essaadaoui et al. [10] studied the adsorption of Co^2+^ ions in wastewater using eucalyptus bark grafted with acrylic acid. The study found that the adsorption process followed a second-order kinetic model. A novel photocatalyst and adsorbent material, copper (II) oxide (CuO)/barley straw-derived silicon dioxide (SiO_2_), has been introduced [11]. The material has demonstrated high efficiency in removing methylene blue from water. To investigate the kinetics of the adsorption process, pseudo first- and second-order models were employed. The results indicate that the pseudo-second-order model provides the best explanation for the behavior of methylene blue adsorption on the nanocomposite. The findings of this study suggest that CuO/barley-straw-derived SiO_2_ material is a promising candidate for removing organic pollutants from water [11]. A hybrid chitosan-derived mesoporous spongy carbon bio-adsorbent (HCMSC) was developed to effectively eliminate iron(III) (Fe^3+^) toxins from water samples for human consumption [12]. The kinetics of Fe^3+^ adsorption were investigated using semiempirical models, among which Elovich’s kinetic model effectively explained the adsorption process. The adsorption process was determined to conform to the pseudo-second-order kinetic model, and the limiting factor is intraparticle diffusion [7]. As reported in [13], a composite material composed of montmorillonite, magnetic nickel ferrite (NiFe_2_O_4_), and amine-functionalized chitosan was developed to serve as a green adsorbent for removing cationic methylene blue and crystal violet dyes from neutral wastewater. The adsorption behavior of this material was analyzed, and it was observed that the process followed the pseudo-second-order model. Additionally, a model was used to investigate the interaction and rate-limiting step between the adsorbent and adsorbate. According to the article [14], a new photocatalyst with low-cost and high efficiency in eliminating crystal violet and producing crystal violet-free water is introduced. This innovative photocatalyst combines zinc oxide (ZnO) nanosheets with silica-fume-derived SiO_2_, resulting in a unique mesoporous ZnO/silica-fume-derived SiO_2_ photocatalyst. Furthermore, the researchers utilized pseudo-first- and second-order kinetics models to study the photodegradation process and found that the degradation of the ZnO/silica-fume-derived SiO_2_ nanocomposite follows the pseudo-first-order model [14]. Salama et al. [15] utilized a sol-gel method to prepare a solid acid substance of mobil composition of matter no. 41 (MCM-41), which was then used to incorporate titanium dioxide (TiO_2_) within the mesochannels through a simple and effective impregnation method. The kinetic models used to investigate the adsorption mechanism were the pseudo-first-order and the pseudo-second-order kinetic models and the intraparticle diffusion models. The pseudo-second-order adsorption mechanism was predominant, suggesting that the adsorption process is controlled by chemisorption involving valency forces through sharing or exchanging electrons between the solvent and the adsorbate.

The intraparticle diffusion model was further analyzed to clarify the diffusion mechanism, which showed that the adsorption process was controlled by film diffusion. The study’s findings are significant for water treatment, especially for removing dye contaminants such as methylene blue. The present study by Bakry et al. [16] investigated the preparation of cerium oxide partially reduced the graphene oxide (CeO_2_-PRGO) nanocomposite and its applications as an efficient adsorbent to remediate water-containing phosphate. The pseudo-second-order kinetic model well fitted the experimental data with a correlation coefficient (R^2^) of over 99%, suggesting the adsorption of phosphate ions onto the surface of CeO_2_-PRGO nanocomposite takes place through the generation of a monolayer of phosphate ions onto the surface of CeO_2_-PRGO. The adsorption mechanism was suggested to be an interaction between phosphate ions and Cerium(3+) (Ce^3+^) sites on the surface of ceria nanoparticles on the adsorbent surface. Research by Ibrahim et al. [17] found that adsorption capacity (q_e_) and R^2^ values were very small compared with those obtained from the pseudo-second-order kinetics. These results revealed that the predominant mechanism is pseudo-second order. In addition, Ibrahim et al. introduced Boyd film diffusion and intraparticle diffusion plots to investigate the rate-limiting step. They found that the adsorption process was divided into two key steps in the case of the intraparticle diffusion model. First, the diffusion of adsorbate molecules into the interior pores of the adsorbent and, second, the migration of solute molecules from the aqueous solution to the surface of adsorbent particles. They concluded that intraparticle diffusion does not control the adsorption mechanism since the rate-limiting step is controlled by intraparticle diffusion when the plot passes through the origin. Therefore, the adsorption mechanism was also controlled by film diffusion [17].

Although numerous researchers have employed chemical reaction models to determine the mass-transfer controlling step of metal-ions adsorption onto various adsorbents, there has been relatively little focus on identifying the rate-limiting steps of diffusion processes, especially when using nanocellulose obtained from waste palm leaves as an adsorbent. Therefore, this study aimed to examine the adsorption of cobalt ions on prepared nancellulose derived from Saudi waste palm leaves using reaction and diffusion models and determine the optimal kinetic model while identifying the rate-limiting step. The study calculated the parameters of various reaction models, including pseudo-first-order, pseudo-second-order, and Elovich, as well as the intraparticle diffusion model. The primary focus of this research was to explore the potential of nanocellulose as an adsorbent for removing cobalt ions from wastewater.

The utility of this study lies in its contribution to developing efficient and ecofriendly methods for treating industrial wastewater that contains high concentrations of heavy metals. The results show that the nanocellulose produced from Saudi waste palm leaves effectively removes cobalt ions from wastewater with excellent kinetics. Additionally, the study offers valuable insights into the adsorption process mechanisms, which can be utilized to enhance process parameters for future applications.

In general, this research has practical implications for creating sustainable solutions for wastewater treatment, particularly in industries such as mining, metallurgy, and electroplating. The findings of this study could help decrease the environmental impact of these industries and enhance the quality of water resources.

This study focused on a new source for extracting nanocellulose derived from Saudi waste palm leaves and used as an adsorbent to remove cobalt ions from wastewater. Unlike previous studies that utilized wastepaper, cotton, and sugarcane bagasse as sources of nanocellulose, this study’s approach is unique and significant. It provides a sustainable and cost-effective method for producing nanocellulose for cobalt-ion adsorption and offers valuable insights into the adsorption mechanism and kinetics. The study analyzes the multiple chemical reactions and mass-transfer processes involved in adsorption, which needed to be fully understood in previous studies. Moreover, by determining the kinetic parameters using tested models, the analysis can contribute to developing theoretical models for predicting the adsorption behavior of nanocellulose. This information is crucial for optimizing the adsorption process and developing effective methods for wastewater treatment. Overall, this study stands out from the previous literature in its use of a novel source material and its focus on addressing cobalt-ion contamination in wastewater.

## 2. Experimental Work

### 2.1. Nanocellulose Biopolymer as an Adsorbent

High-grade laboratory chemicals, including potassium hydroxide (KOH), sulfuric acid (H_2_SO_4_), acetic acid (CH_3_COOH), and sodium chlorite (NaClO_2_) were procured from BDH chemicals for use in the project. Palm leaf waste from Al-Karaj City, Saudi Arabia was ground in a planetary ball mill at 250 rpm for 6 h to obtain a powder of <65 µm. The powder was mixed with distilled water and sonicated using an SFX550 (Sonifier, Suwanee, GA, USA) at 480 W for 70 min. Sulfuric acid was then slowly added to the mixture while heating to 90 °C. The mixture was centrifuged at 3000 rpm for 20 min, the residue was filtered and washed several times with deionized water to neutral pH. Afterward, hydrolysis treatment was performed at 90 °C for 4 h using 100 g of distilled water and 5 g of potassium hydroxide to remove lignin. It was then filtered and washed with deionized water to ensure the purification of the residue and obtain a neutral pH. In this method, cellulose is treated with a sodium chlorite solution (4 g/100 g water) to remove amorphous cellulose, and the solution is stirred at a fixed temperature (80 °C) for 6 h. The final product was filtered and neutralized by washing it several times with deionized water. The residue was then dried in an oven at 90 °C until a constant weight was reached, then sulfuric acid (50% by weight) was added at 50 °C for 70 min. After that, the suspension was washed by centrifugation again at 3000 rpm for 20 min, sonicated at 480 W for 20 min, then centrifuged at 3000 rpm for 20 min and filtered. To obtain nanocellulose as a biopolymer with an average size of 85 nm, the precipitate needs to be collected and dried in an oven at 50 °C for 4 h, followed by TEM (200,000×) analysis of the nanocellulose.

### 2.2. Characterization of Nanocellulose Biopolymer as an Adsorbent

#### 2.2.1. Transmission Electron Microscopy (TEM)

Transmission electron microscopy (TEM), (JEM-2100F, JEOL Ltd., Tokyo, Japan), at an accelerating voltage of 200 kV, was used for the prepared nanocellulose samples. Typically, samples are sonicated in ethanol for 6 h, then spotted onto carbon-coated grids and allowed to dry overnight. This process ensures that the sample is evenly distributed within the grid and is fully prepared for imaging. 

#### 2.2.2. Fourier Transform Infrared Spectroscopy (FT-IRS)

FT-IRS studies were performed in the wavelength range of 400–4600 cm^−1^ using a mixture of 4 mg prepared nanocellulose powder and 200 mg KBr. The spectra were obtained by the coaddition of individual scans using an FT-IRS device (ALFUAS, BRUKER type, Bruker Corporation, Billerica, MA, USA) and a scan resolution of 4 cm^−1^.

#### 2.2.3. BET Surface Area

Gas adsorption–desorption techniques for determining the parameters of porous materials use the concepts of Brunauer–Emmett–Teller (BET) and Barret, Joyner, and Halenda (BJH). This procedure determines the pore size of the prepared nanocellulose. Estimation of the pore-size distribution of nanocellulose using a Micrometric ASAP-2020 (ASAP-2020 microporous analyzer, Micromeritics Instrument Corporation, Norcross, GA, USA). Before testing, the nanocellulose was vacuum degassed at 80 °C for 18 h. The pore size of the nanocellulose was determined by a nitrogen adsorption–desorption technique at 77 K using the Brunauer–Emmett–Teller (BET) method.

#### 2.2.4. Zeta Potential

Brookhaven Instruments Corporation, an industrial equipment manufacturer, produces Zeta Potential Analyzers. The device (Zetasizer Ver. 7.10, Malvern Instruments Ltd., Worcestershire, UK) was used to determine the zeta-potential change and surface charge of the prepared nanocellulose. Additionally, the change in zeta potential versus pH was studied to determine the adsorbent surface charge.

#### 2.2.5. X-ray Diffraction

The X-ray diffraction technique (diffractometer used: model D8AD VANCE, BRUKER, Billerica, MA, USA) was used to identify the crystalline phases of the nanocellulose sample. In this technique, the scattered intensity of an X-ray beam, generated upon hitting the nanocellulose sample, is measured as a function of the incident angle. The nanocellulose sample for XRD analysis was dried for 24 h in a vacuum oven at 105 °C to eliminate any moisture present in the material. Then, the nanocellulose sample was powdered and spread on the glass holder with a gap of 0.5 mm. The holder with the sample was then placed in the X-ray chamber and scanned at a constant temperature and a speed of 2/min using CuKα radiation, over a diffraction angle (2θ) range from 10° to 60°, with a step size of 10°. The Joint Committee on Powder Diffraction Standards (JCPDS) diffraction file cards (2001) are used as a reference for the interpretation of the X-ray patterns obtained in the experiment.

### 2.3. Experimental Adsorption Equilibrium Isotherm

To investigate the impact of cobalt-ion concentration on adsorption capacity, isotherm experiments were conducted using 85 nm particles with varying initial cobalt solution concentrations at a temperature of 20 °C. The adsorption process reached equilibrium after approximately 90 min, and adsorbed cobalt-ion concentrations were determined using the equation:

q_e_ = V (C_o_ − C_e_)/m (1)

**Figure 1:**
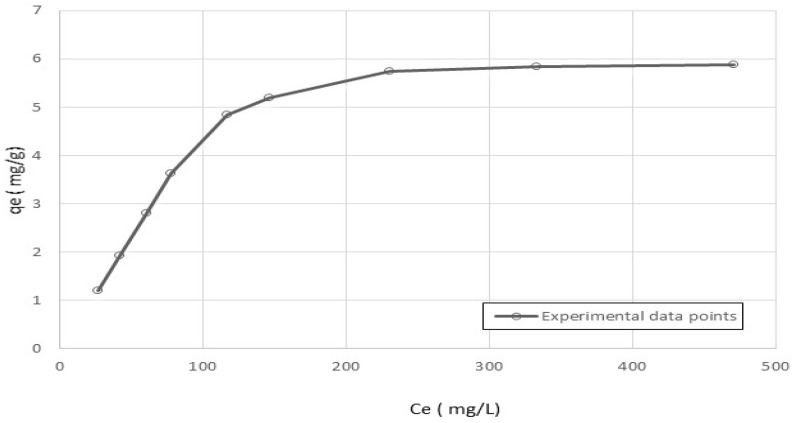
Adsorption of cobalt ions on nanocellulose at 20 °C.

Figure 1 displays the resulting equilibrium adsorption isotherm curve, which plots the amount of cobalt adsorbed on the nanocellulose against the cobalt equilibrium concentration in the solution.

### 2.4. Kinetic Experiments

Initial cobalt-ion concentrations were measured in special bottles placed in a thermostat-controlled shaker. This allowed for precise and accurate measurement of the adsorption rate when performing kinetic experiments. For example, 90 min of adsorption measurements were performed using a specific agitation speed. First, one gram of nanocellulose was added to 50 mL of cobalt-ion solution. After this, the adsorption rates decreased until equilibrium was reached. Bottle samples were taken at various times during shaking for 90 min. After filtering the liquid, an atomic spectrometer (PerkinElmer AAnalyst 700 atomic spectrometer, PerkinElmer Inc., Massachusetts, USA) measured the amount of cobalt in the solution.

Experiments were conducted to evaluate the effect of agitation speed and initial concentration on the adsorption rate of 1 g of nanocellulose (84.2 nm particle diameter) in 50 mL of cobalt-ion solution (200 ppm, pH = 5) at 20 °C.

**Figure 2:**
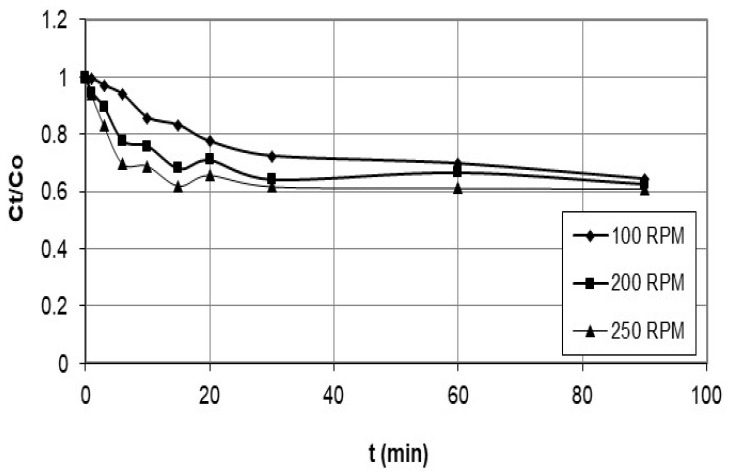
Adsorption rate of cobalt ions on nanocellulose at C_o_ = 200 ppm and varying stirrer speeds.

In the adsorption process, the agitation speed plays a crucial role in mixing the solution, the mass transfer of the adsorbate to the adsorbent, and the diffusion of the adsorbate into the adsorbent’s pores. A range of speeds is typically tested to investigate the effect of agitation speed on the adsorption kinetics. The range of agitation speeds is usually determined from previous literature, such as the study by Kyzas et al. [18], which examined the effect of agitation speed on nanobubble-facilitated batch adsorption using speeds of 150 to 225 rpm. Once a range of agitation speeds is selected, various speeds are evaluated to determine their impact on the adsorption kinetics. Therefore, the specific agitation speeds of 100 rpm, 200 rpm, and 250 rpm were tested in this study and were used to investigate the effect of agitation speed on the adsorption kinetics, and the initial cobalt concentration was measured before adding the nanocellulose. The cobalt solution was sampled and filtered at different periods, and the concentration was determined by atomic absorption spectrometry. The data was plotted as concentration vs. time, as shown in Figure 2.

**Figure 3:**
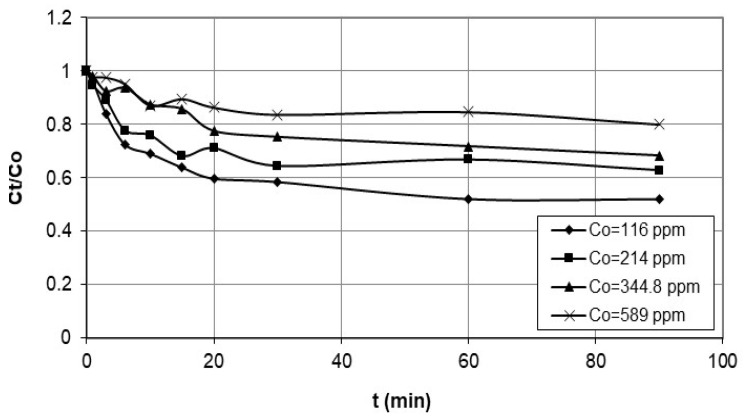
Adsorption rate of cobalt ions on nanocellulose at 200 rpm and varying initial concentrations.

Moreover, the tests were conducted with initial concentrations ranging from approximately 100 ppm to 600 ppm, while maintaining an agitation speed of 200 rpm and identical solution conditions for both nanocellulose and cobalt. The data collected was plotted as concentration vs. time, as shown in Figure 3. As per the findings of Quiton et al. [19], cobalt concentrations in real electroplating wastewater generally vary from 3 to 1500 mg/L. Therefore, the cobalt concentrations utilized in this study fall within the cobalt concentrations commonly observed in electroplating wastewater.

## 3. Adsorption Equilibrium Isotherm Models

The experimental results for the removal of cobalt ions by nanocellulose are described using two isotherm models. The Langmuir and Freundlich schemas are such models.

### 3.1. Langmuir Isotherm Model

The Langmuir equation used to determine the maximum capacity of nanocellulose assumes that metal-ion adsorption on the material occurs in a monolayer. Here is the Langmuir isotherm equation:

(2) $$q_{e}=\frac{K\;C_{e}}{1+bC_{e}}$$

The Langmuir model in the linear form is:

(3) $${\frac{C_{{}_{e}}}{q_{e}}}_{}=\frac{1}{K_{}}+\left(\frac{b}{K_{}}\right){C_{e}}_{}$$

Hence, a plot of C_e_/q_e_ versus C_e_ provides the equilibrium-constant parameters and *K* and *b*, for the adsorption process from the slope and intercept of the linear plot. 

### 3.2. Freundlich Isotherm Model

The Freundlich isotherm model was used to describe the experimental data for heterogeneous surfaces. This is the format for the Freundlich form:

(4) $$q_{e}=K_{F\;\;}C_{e}^{1/n}$$

The Freundlich model in the linear form is:

(5) $$\log\;q_{e}=\log\;K_{F}+\left(\frac{1}{n}\right)\;\log\;C_{e}$$

Hence, a linear plot of log q_e_ versus log C_e_ provides the equilibrium-constant parameters, *n* and K_F_, for the adsorption process.

## 4. Kinetic Models

Kinetic models are described, including those in which chemical reactions are the rate-limiting step to explain experimental results, and in which mass-transfer processes are the rate-limiting step to explain the results.

### 4.1. Describing the Batch Adsorption Process Using Reaction Models

Many researchers have used the pseudo-first-order, pseudo-second-order, and Elovich models to analyze the adsorption of heavy-metal ions in chemical reactions that operate as rate-controlling mechanisms [7,8,9,10,11,12,13,14,15,16,17].

#### 4.1.1. Pseudo-First-Order Model

The first-order kinetic equation of the batch process in which the adsorption rate of cobalt ions on nanocellulose is proportional to the amount of cobalt adsorption can be expressed as:

(6) $$\left(\frac{{\mathrm{dq}}_{t}}{\mathrm{dt}}\right)=k_{1}\;(q_{e}-q_{t})$$

Integrate Equation (6), where q_e_ and q_t_ are the adsorption capacities (mg/g) of cobalt ions at equilibrium and time t, respectively, and k_1_ is the pseudo-first-order adsorption-rate constant (1/min), and use the initial condition q_t_ = 0 at t = 0, resulting in:

ln (q_e_ − q_t_) = ln q_e_ − k_1_ t (7)

Here, q_t_ can be found using the following method:

(8) $$q_{t}=\frac{V_{}\left(C_{o}-C_{t}\right)}{M}$$

In addition, q_e_ can be calculated as follows:

(9) $${}_{}q_{e}=\frac{V_{}\left(C_{o}-C_{e}\right)}{M}$$

where:

C_o_ = initial concentration of cobalt ions (mg/L)

C_t_ = concentration of cobalt ions at time t (mg/L); 

C_e_ = concentration of cobalt ions at equilibrium (mg/L).

#### 4.1.2. Pseudo-Second-Order Model

A modified second-order pseudoequation describes the cobalt adsorption. It can be written as:

(10) $$\left(\frac{{\mathrm{dq}}_{t}}{\mathrm{dt}}\right)=k_{2}\;(q_{e}-q_{t}{)}^{2}$$

By using the adsorption capacity of cobalt ion at equilibrium (q_e_), and at a specific time (q_t_), in mg/g, along with the pseudo-second-order adsorption-rate constant (k_2_) in g/mg·min, Equation (10) can be integrated, and the boundary condition of q_t_ = 0 at t = 0 and q_t_ = q_t_ at t = t can be applied.

(11) $$\left(\frac{t_{}^{}}{\overset{}{q_{t}^{}}}\right)=\left(\frac{1}{\overset{}{k_{2}q_{e}^{2}}}\right)+\left(\frac{1}{\overset{}{q_{e}^{}}}\right)t$$

A graph of (t/q_t_) versus t for Equation (11) should exhibit a linear relationship with a slope of 1/q_e_ and a y intercept of (1/$k_{2}^{}q_{e}^{2}$).

#### 4.1.3. Elovich Model

The Elovich equation is expressed as follows:

(12) $$\left(\frac{{\mathrm{dq}}_{t}}{\mathrm{dt}}\right)\;=\;œ\;\exp\;(-ß\;q_{t})$$

where it is the cobalt amount adsorbed on nanocellulose at time t, œ is the initial cobalt adsorption rate (mg/g·min), and ß is the desorption constant (g/mg) for a given experiment period. For the simplification of the Elovich equation, assuming ß > 1, the boundary condition q_t_ = 0 is applied when t = 0, and q_t_ = q_t_ is applied when t = t, then Equation (12) becomes [20]:

q_t_ = ß ln (œ ß) + ß ln t (13)

To determine the Elovich equations that fit the kinetics, Equation (13) was used to represent the adsorption rate of cobalt on nanocellulose. Thereafter, constant values can be obtained from the linear slope and intercept of the plot of q_t_ versus lnt.

### 4.2. Describing the Batch Adsorption Process Using an Intraparticle Diffusion Model

In general, diffusion models that describe the adsorption process include an external mass-transfer coefficient and internal pore since the reaction rate is rapid compared to the mass-transfer steps [17,21]. Therefore, external and internal diffusion resistance will control the adsorption-rate process. Thus, the intraparticle diffusion model is used if mass-transfer processes operate as the rate-controlling mechanism.

Intraparticle models are used to determine macropore mass-transfer coefficients when the internal mass transfer controls the adsorption rate. The model assumes that pore diffusion is the rate-limiting step. It can be used to interpret experimental adsorption data and estimate kinetic parameters such as macropore-diffusion coefficients. This can then be used to predict adsorption behavior in batch adsorbers. Pore diffusion, or the diffusion of ions within the pores of the sorbent, is a key component of the model. When applying intraparticle models to adsorption processes governed by internal mass transfer, the assumptions suggested by McKay [22,23] must be made.

- The initial cobalt concentration is evenly distributed in the total solution. In contrast, the cobalt concentration on the outer surface is zero at the beginning of the adsorption process (t = 0);
- There is a local equilibrium between the cobalt concentration in the adsorbent pores and the cobalt adsorption on the inner pore-surface sites. Therefore, the linear-equilibrium isotherm equation can be applied as an adsorption equilibrium equation;
- Compared to the internal resistance to mass transfer, the external mass-transfer resistance’s effect is negligible;
- Adsorbent particles take the shape of spherical particles;
- The diffusion coefficient is constant and cobalt diffusion occurs only along the radial axis of a spherical particle.

Based on the given assumptions, the linear-equilibrium adsorption isotherm gives a linear parabolic partial-differential equation for the cobalt mass balance on the adsorbent particles.

∂C∕∂t = D_p_ (∂^2^C∕∂r^2^ + 2/r ∂C∕∂r) (14)

with boundary conditions:

C_r,0_ = C_o_ for r = R,  t > 0

C_r,∞_ = C_∞_ for 0 ≤ r ≤ R, t > 0

where: 

C_o_ = the initial concentration of the cobalt and C_r_ concentration of cobalt on the particle’s outer surface, no external mass-transfer resistance was assumed.

Solving the linear parabolic partial-differential Equation (14) using the separation-of-variables method or the Laplace transform method gives the following formula [24]:

(15) $$q_{t}/q_{m}=6\;(D_{p}\;t/R^{2}{)}^{1/2}\;\{\pi^{-1/2}+2\;\sum_{n=1}^{\infty }\;\mathrm{ierfc}\;\mathrm{nr}/\left(Dp\;t\right)\overset{1/2}{}\;\}-3\;D_{p}\;t/R^{2}$$

where:

q_t_: the mass of cobalt adsorbed per mass of nanocellulose in the internal pores time t;

q_m_: adsorption of cobalt in the inner pores of nanocellulose;

q_t_ and q_m_ are related to the cobalt concentrations in the solution and the pores by the linear-equilibrium relationship i.e., q_t_ = K (C_o_ − C_t_) and q_m_ = K (C_o_ − C_e_). 

Equation (15) shows that the initial experimental adsorption data form a straight line when plotting q_t_/q_e_ on the y-axis and t^0.5^ on the x-axis. This relationship is clearly shown.

Since the relationship between q_t_/q_m_ versus the square root of time holds only for short times, i.e., D_p_t/R^2^ < 1, it is important to obtain adsorption data during the initial period of adsorption before the higher-order terms in the above equation become important. The adsorption data, however, must be accepted after the short period that is controlled by external mass transfer. 

The macropore rate parameter, D_p_, can be calculated from the slope of the line formed when q_t_/q_m_ is plotted against t^0.5^ [22].

## 5. Results and Discussions

### 5.1. Nanocellulose as Biopolymer Characterizations

**Figure 4:**
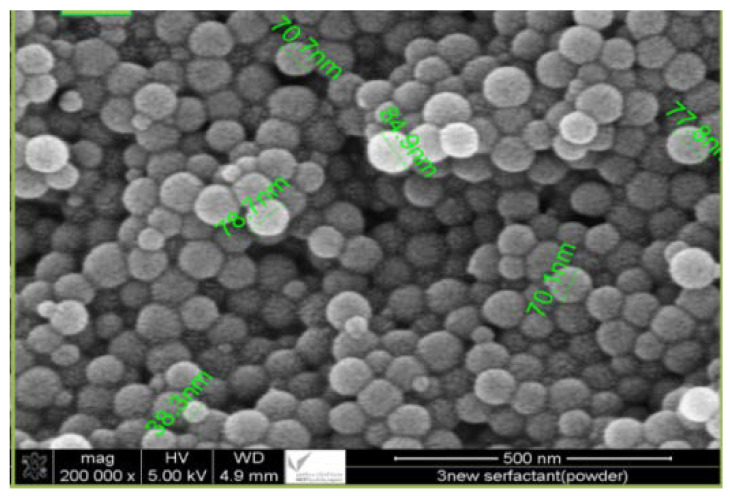
TEM of the nanocellulose (200,000×).

Figure 4 shows the transmission electron microscopy (TEM) of the prepared nanocellulose (200,000×), where the nanocellulose have an average size of 85 nm.

**Figure 5:**
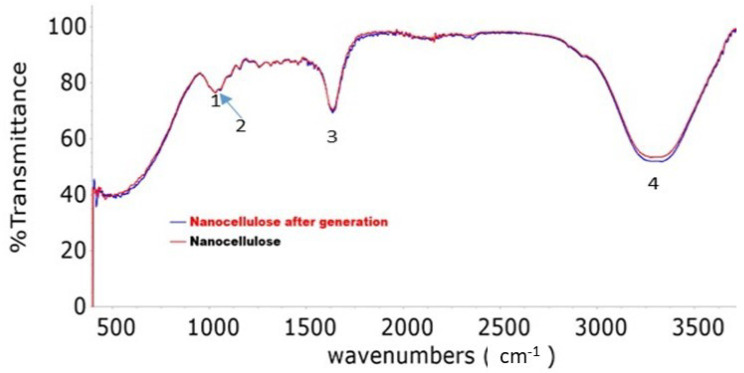
FT-IR spectrum of nanocellulose. Peak 1 (1027 cm^−1^): C-O stretching in cellulose. Peak 2 (1164 cm^−1^): C-O stretching in cellulose and hemicellulose. Peak 3 (1730 cm^−1^): C=O stretching in hemicellulose. Peak 4 (3000–3399 cm^−1^): CH stretching in cellulose, hemicellulose.

Figure 5 illustrates the peaks in the FT-IR spectrum of the scanned nanocellulose sample, with peaks appearing at 1027 to 1730 cm^−1^ and 3000 to 3399 cm^−1^, indicating the presence of cellulose bonds. 

**Figure 6:**
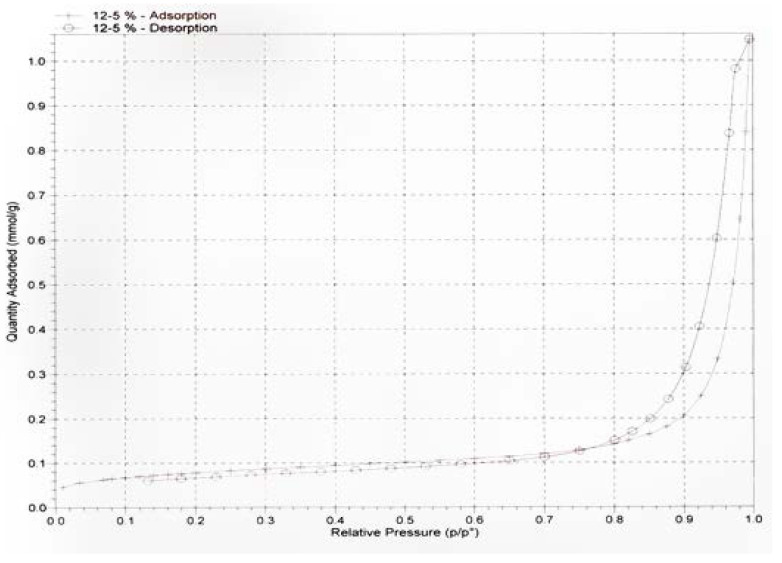
Nitrogen adsorption–desorption isotherms.

The pore size of the nanocellulose was determined by a nitrogen adsorption–desorption technique at 77 K, as shown in Figure 6. Nitrogen adsorption–desorption isotherms were performed using the Brunauer–Emmett–Teller (BET) method to determine the nanocellulose pore size. The data derived from the isotherms are listed in Table 1.

**Figure 7:**
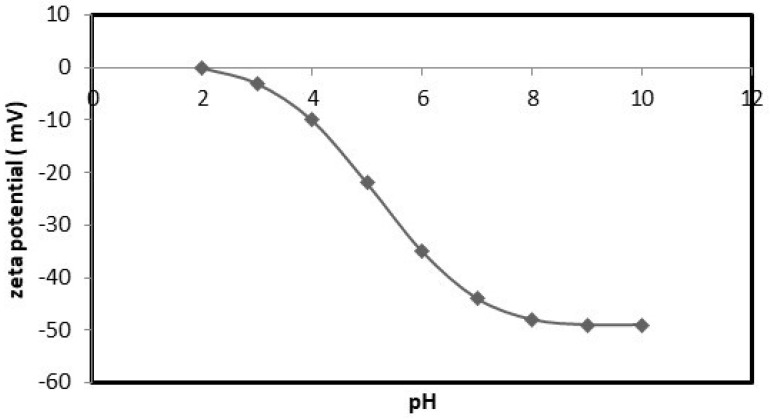
Variation of zeta potential versus pH for the nanocellulose.

The zeta-potential curve of the nanocellulose is presented in Figure 7. The isoelectric point of the nanocellulose appears at pH 2, where the zeta potential was negative. 

**Figure 8:**
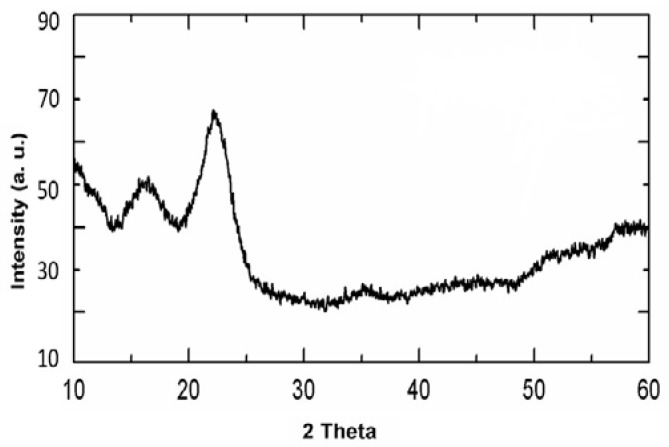
XRD for nanocellulose.

By subjecting date palm leaves to a series of chemical treatments, cellulose nanocrystals (CNCs) were successfully extracted. The identification of specific diffraction peaks in the XRD data (Figure 8) confirmed the presence of CNCs and allowed for the determination of the crystallinity of the samples. The crystal structure of the CNCs was characterized by two distinct peaks, located at 2θ = 22.3° and 2θ = 16.3°, respectively [25].

### 5.2. Adsorption-Equilibrium Isotherm

The adsorption capacity was determined using Equation (1), which relates the adsorbed amount (q_e_) to the initial and equilibrium concentrations of cobalt ions in the solution, the mass of the adsorbent (m), and the volume of the solution (V). The resulting data were plotted in Figure 1, which shows the equilibrium-adsorption isotherm curve. This curve illustrates the relationship between the amount of cobalt adsorbed on the nanocellulose particles and the equilibrium concentration of cobalt ions in the solution. The maximum adsorption amount of cobalt ions at room temperature was found to be 5.98 mg/g, which represents the highest amount of cobalt ions that can be adsorbed per unit mass of nanocellulose particles under these experimental conditions.

The experimental results for the removal of cobalt ions by nanocellulose are described using two isotherm models. The Langmuir and Freundlich schemas are such models. The equilibrium parameters were obtained by fitting the isotherm models with the equilibrium experimental data.

The Langmuir model was used to plot the C_e_/q_e_ versus C_e_ to provide the equilibrium-constant parameters and *K* and *b* for the adsorption process from the slope and intercept of the linear plot.

**Figure 9:**
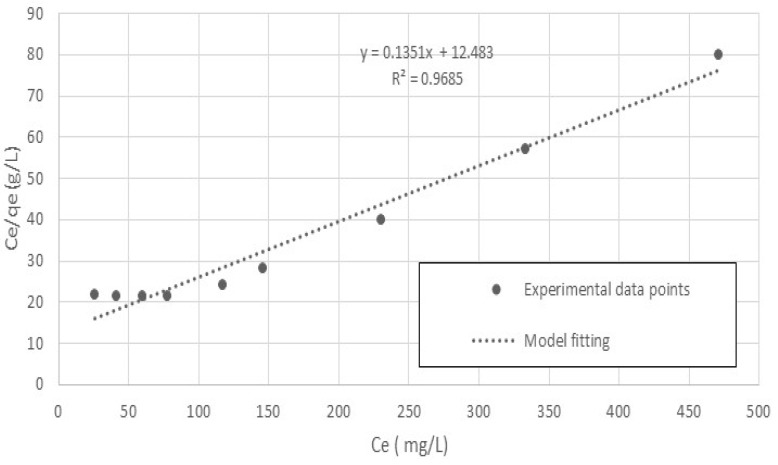
Langmuir isotherm fitting the experimental data of cobalt ion adsorption onto nanocellulose.

Figure 9 shows the linear relationship between C_e_/q_e_ and C_e_ for T = 20 °C. The equilibrium parameters, *K* and *b* of the system were determined and are listed in Table 2. According to the value of the adjusted coefficient of determination (R^2^), as shown in Figure 9, the Langmuir model adequately explained the experimental data.

**Figure 10:**
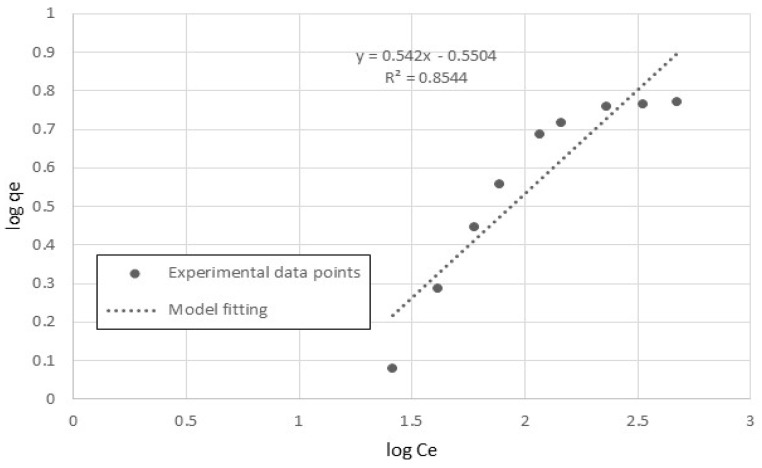
Freundlich isotherm fitting the experimental data of cobalt-ion adsorption onto nanocellulose.

The Freundlich model was used to plot log q_e_ versus log C_e_ to provide the equilibrium-constant parameters, *n* and K_F_, for the adsorption process from the slope and intercept of the linear plot. Figure 10 shows the linear relationship between log q_e_ and log C_e_ at T = 20 °C. The equilibrium parameters, *n* and K_F_ of the system were determined are listed in Table 2. The value of *n* is greater than one, which may indicate that cobalt ions are readily adsorbed by nanocellulose [26].

From Figure 10, we can see that the Freundlich model fits the experimental data well. This is further supported by the coefficient of determination (R^2^) values.

As shown by the (R^2^) values in Table 2, the Langmuir model can more accurately fit the adsorption experimental data of cobalt ions on nanocellulose than the Freundlich model.

### 5.3. Kinetic Studies

Figure 2 and Figure 3 describe a set of experiments conducted to investigate the effect of agitation speed and initial concentration on the adsorption rate of nanocellulose in a cobalt-ion solution. Agitation speed is a crucial factor in the adsorption process, as it affects the mixing of the solution, the transfer of the adsorbate to the adsorbent, and the diffusion of the adsorbate into the pores of the adsorbent. A range of agitation speeds were tested in this study, and speeds of 100 rpm, 200 rpm, and 250 rpm were selected for evaluation.

The initial cobalt concentration was measured before adding the nanocellulose, and the cobalt solution was sampled and filtered at different time intervals to determine the concentration using atomic-absorption spectrometry. The data collected was plotted as concentration vs. time. The experiment was also conducted with different initial concentrations ranging from approximately 100 ppm to 600 ppm while maintaining a constant agitation speed of 200 rpm and identical solution conditions for both the nanocellulose and the cobalt. The data collected was also plotted as concentration vs. time.

In the case of the adsorption of cobalt ions using nanocellulose as the adsorbent, the concentration of cobalt ions in the wastewater would initially be higher than the concentration on the nanocellulose surface. As the cobalt ions come into contact with the nanocellulose surface, they start to attach to the surface of the nanocellulose through electrostatic interaction mechanisms.

As more and more cobalt ions attach to the nanocellulose surface, the concentration of cobalt ions in the wastewater decreases. This leads to a decrease in the concentration of cobalt ions in the wastewater with time, as observed in the plot of concentration vs. time.

The rate of decrease in the concentration of cobalt ions in the wastewater will depend on factors such as the initial concentration of cobalt ions, and the amount and surface area of the nanocellulose adsorbent used in the wastewater. The equilibrium concentration of the cobalt ions in the wastewater will eventually be reached when the rate of attachment of cobalt ions to the nanocellulose surface is equal to the rate of detachment of cobalt ions from the nanocellulose surface.

The adsorption of cobalt ions onto the nanocellulose from wastewater is influenced by several factors, including the initial concentration of cobalt ions in the wastewater. Generally, it can be observed that the lower initial concentrations of cobalt ions decrease faster and at a higher rate than the higher initial concentrations during the adsorption process.

This is due to the adsorption process that occurs through the binding of cobalt ions onto the surface of the nanocellulose, where the concentration of available binding sites is limited. At low initial concentrations of cobalt ions, there are more available binding sites on the nanocellulose surface for the cobalt ions to attach to. As a result, the rate of adsorption of cobalt ions onto the nanocellulose surface is higher at lower initial concentrations. On the other hand, at higher initial concentrations of cobalt ions, there are fewer available binding sites on the nanocellulose surface, as the available sites are already occupied by cobalt ions. This means that as the adsorption process continues, the rate of adsorption decreases as the number of available binding sites decreases, resulting in a slower decrease in concentration over time. Furthermore, at higher initial concentrations of cobalt ions, the concentration gradient between the wastewater and the nanocellulose surface is higher, which leads to a slower rate of adsorption due to a lower driving force for the adsorption process to occur. Therefore, the rate of decrease in concentration during the adsorption of cobalt ions onto nanocellulose is influenced by the initial concentration of cobalt ions in wastewater, with lower initial concentrations leading to a faster rate of decrease in concentration [27].

Agitation speed can affect the rate of adsorption by influencing the transport of cobalt ions from the bulk solution to the surface of the nanocellulose. At high agitation speeds, the fluid motion around the nanocellulose surface is increased, which leads to an increased rate of mass transfer and diffusion of cobalt ions to the nanocellulose surface. The increased fluid motion can help to maintain a higher concentration gradient between the bulk solution and the nanocellulose surface, leading to a faster rate of adsorption. On the other hand, at low agitation speeds, the transport of cobalt ions from the bulk solution to the nanocellulose surface is limited, and mass transfer can become the rate-limiting step in the adsorption process. As a result, the rate of adsorption can be slower. Therefore, higher agitation speeds can lead to faster and more efficient adsorption of cobalt ions onto nanocellulose, resulting in a faster decrease in cobalt-ion concentration over time [28].

#### 5.3.1. Investigation of the Mechanism of Adsorption Using Experimental Kinetic Data

The objective of this study was to investigate the adsorption behavior of cobalt ions on nanocellulose and analyze the kinetic parameters of the adsorption process to gain a better understanding of the adsorption mechanism. To achieve this goal, the study employed several kinetic models, such as the pseudo-first-order, pseudo-second-order, Elovich models, and the intraparticle diffusion model. The findings indicated that the agitation speed and initial concentration of cobalt ions had a significant impact on the adsorption rate. Furthermore, the study determined the optimal kinetic model for the adsorption process and concluded that both the general chemical reactions and the diffusion resistance played crucial roles in the removal of cobalt ions.

To gain a more profound understanding of the adsorption mechanism, this study analyzed two scenarios using experimental kinetic data on nanocellulose. These scenarios were based on the study’s findings and aimed to determine the controlling factor in the adsorption process. The first scenario employed several kinetic models, such as the pseudo-first-order, pseudo-second-order, and Elovich models, to investigate the rate of the chemical reaction between cobalt ions and nanocellulose as the primary controlling step. On the other hand, the second scenario utilized the intraparticle diffusion kinetic model to explore the transport of cobalt ions as the dominant factor in the adsorption process. As a result, the study examined two controlling situations: chemical-reaction controlling and cobalt-transport controlling.

##### Discussion of the Effect of the Chemical Reaction as a Rate Controlling Step Using Reaction Models to Describe the Chemical-Reaction Mechanism

Nanocellulose exhibits great potential as an adsorbent material for removing heavy-metal ions from wastewater due to its highly porous and large surface area [29]. Metal ion removal using nanocellulose involves the adherence of metal ions to the surface of the adsorbent material [30]. When nanocellulose is added to wastewater containing heavy-metal ions, the metal ions are adsorbed onto the nanoporous surface of the material due to its high porosity and surface area [29]. The process of adsorption is driven by physical and chemical interactions between the functional groups on the surface of the nanocellulose and the metal ions [30]. To determine the surface charge of nanocellulose as an adsorbent, Figure 7 presents a study of the zeta potential of nanocellulose as a function of pH. The results suggest that the pH of the solution influences the adsorption of cobalt ions onto the nanocellulose. As the pH value increases, the surface charge of the nanocellulose becomes more negative, resulting in the electrostatic attraction of positively charged cobalt ions, leading to their increased adsorption. The removal of cobalt is significantly higher when the pH is increased to pH eight, with an increase in the adsorption quantity until pH seven. Beyond pH seven, cobalt hydrolyzes into Co(OH)^+^, resulting in precipitation in the form of Co(OH)_2_ [8]. Hence, adsorption at pH five is preferred. 

To enhance understanding of the adsorption mechanism, the study utilized multiple kinetic models. These models include the pseudo-first-order, pseudo-second-order, and Elovich models, which are useful for analyzing the adsorption of heavy-metal ions when a chemical reaction controls the rate. 

**Figure 11:**
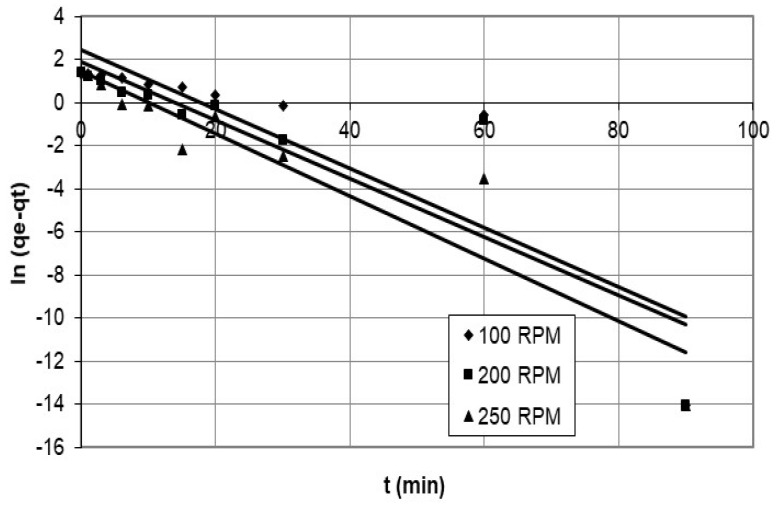
Plots of pseudo-first-order model for adsorption of cobalt ions on nanocellulose at different agitation speeds.

**Figure 12:**
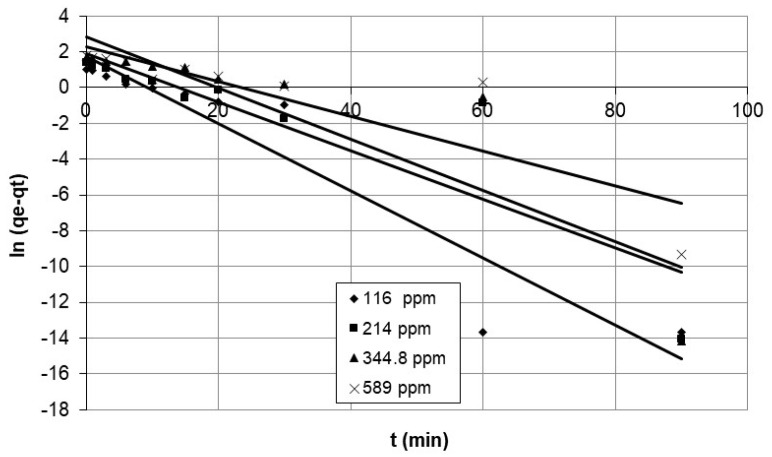
Plots of pseudo-first-order model for adsorption of cobalt ions on nanocellulose at different initial concentrations.

The study focused on the adsorption of cobalt ions on nanocellulose and used the pseudo-first-order model to analyze the data presented in Figure 11 and Figure 12, which show the results for different initial concentrations and agitation speeds. The corresponding parameters are listed in Table 3, and the model accurately depicts the experimental data for the first 15 min.

**Figure 13:**
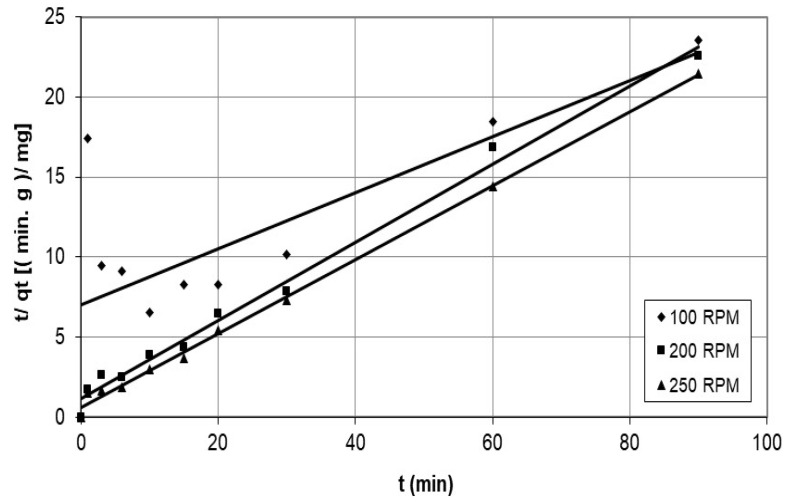
Graphs of the pseudo-second-order model for cobalt ions adsorption on nanocellulose at various agitation speeds.

**Figure 14:**
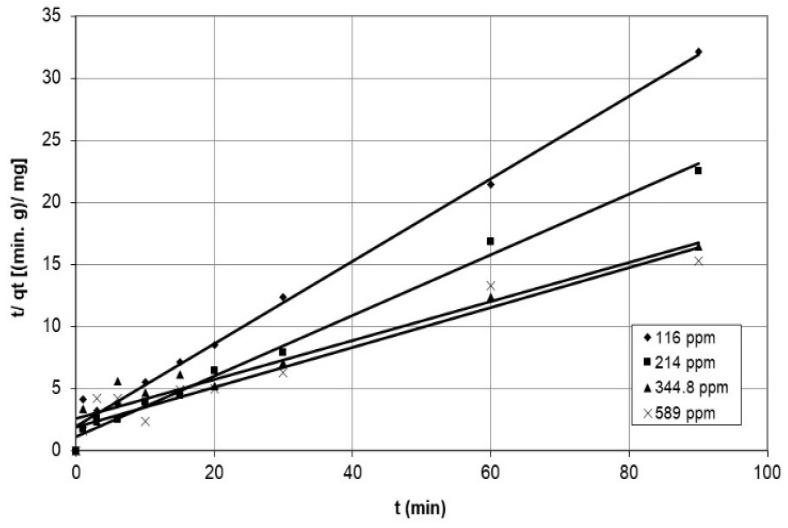
Graphs of the pseudo-second-order model for cobalt ions adsorption on nanocellulose at different initial concentrations.

Furthermore, the study used the pseudo-second-order model to examine the adsorption of cobalt ions on the nanocellulose, and the results are presented in Figure 13 and Figure 14, representing various initial concentrations and agitation speeds. The corresponding parameters are listed in Table 3. The model accurately represents the experimental data, except for the case of an agitation speed of 100 rpm, where the correlation coefficient was 0.577 over a limited time range of 0–3 min, suggesting that the rate-determining process is a chemical reaction.

**Figure 15:**
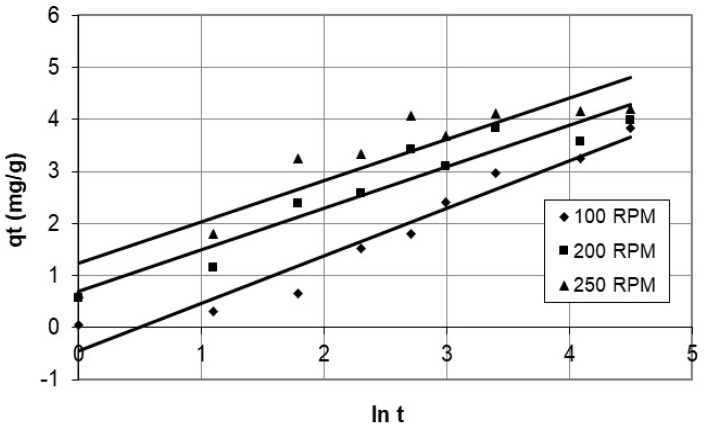
Plots of the Elovich model for adsorption of cobalt ions on nanocellulose at different agitation speeds.

**Figure 16:**
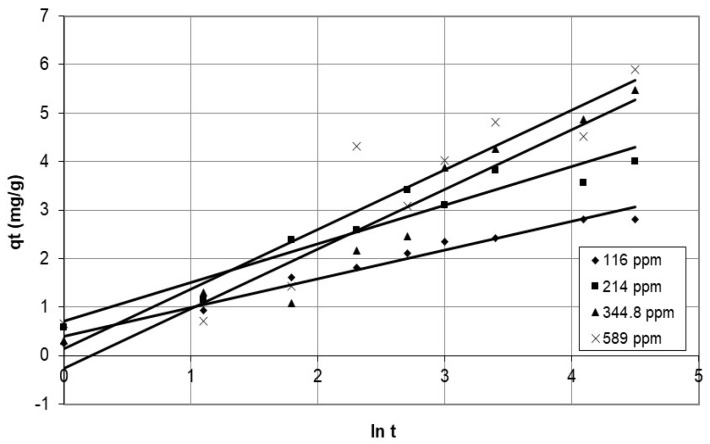
Plots of the Elovich model for adsorption of cobalt ions on nanocellulose at different initial concentrations.

In addition, the study employed the Elovich equation to present the results of cobalt ion adsorption on nanocellulose, as depicted in Figure 15 and Figure 16, for various initial concentrations and agitation speeds. The parameters for the Elovich model are in Table 3. The Elovich model is a good fit for the experimental data, as evidenced by the correlation coefficients, with a minimum value of 0.849. It is suitable for the highly heterogeneous system of cobalt adsorption on nanocellulose and may indicate a chemisorption process [31]. Furthermore, other studies by Averett et al. [32] and Leenheer et al. [33], which used NMR and FT-IR to investigate copper binding with carboxylate groups on humic and fulvic acid, found inner-sphere complex formation via chemisorption, though with reversible outer-sphere binding.

##### Discussion of the Effect of Mass Transfer as a Rate-Controlling Step Using Intraparticle Diffusion Model to Describe Diffusion Mechanism

**Figure 17:**
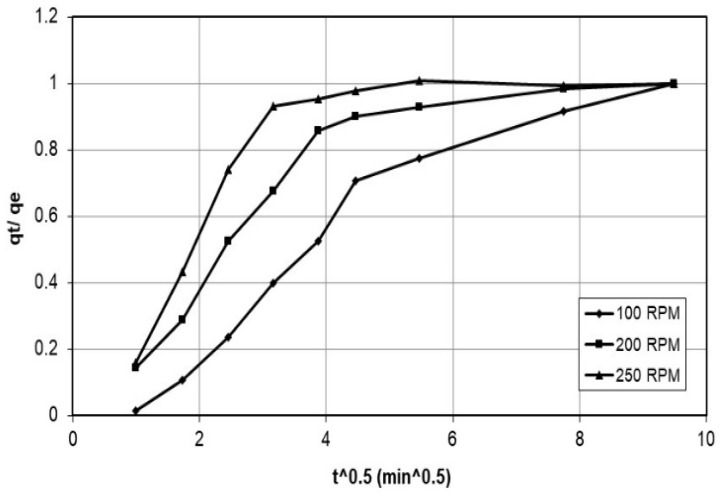
The plot of the intraparticle diffusion model for adsorption of cobalt ions on nanocellulose at different agitation speeds.

**Figure 18:**
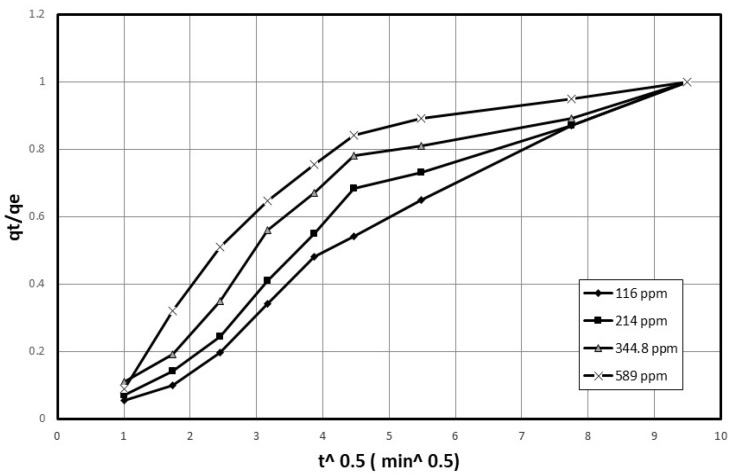
The plot of the intraparticle diffusion model for adsorption of cobalt ions on nanocellulose at different initial concentrations.

The study applied the intraparticle diffusion kinetic model, to better understand the adsorption mechanism. An intraparticle diffusion model is tested at experimental conditions. According to numerous researchers [17,22,23,34,35,36,37], the mechanism of cobalt adsorption on adsorbent can be derived from the plots of q_t_/q_m_ versus t^0.5^. The diffusion of cobalt throughout the adsorbent particle can be described utilizing the relationship between q_t_/q_m_ and t^0.5^, given in the liner section [38]. In addition, the outer-diffusion resistance range can be determined by joining (the extrapolation of) the linear section with the time axis [23]. Figure 17 and Figure 18 show the relationship between q_t_/q_m_, on nanocellulose versus t^0.5^. The macropore diffusion coefficients were calculated and are listed in Table 4. The linear data points, immediately after the initial external mass transfer control period, were used with Equation (15) to calculate D_p_. 

The objective of this study was to investigate the effect of agitation speed and initial concentration on the adsorption rate of nanocellulose in a cobalt-ion solution. Specifically, the intraparticle diffusion parameter for cobalt-ion adsorption on nanocellulose was examined at varying initial concentrations and agitation speeds. The results provide insights into heavy-metal ion adsorption when mass transfer controls the adsorption rate.

Figure 17 displays the relationship between q_t_/q_e_ and t^0.5^ for different agitation speeds. The linear portion of the graph reflects the primary controlling factor of intraparticle diffusion. However, a curve in the graph indicates a decrease in the rate of diffusion [22]. 

**Figure 19:**
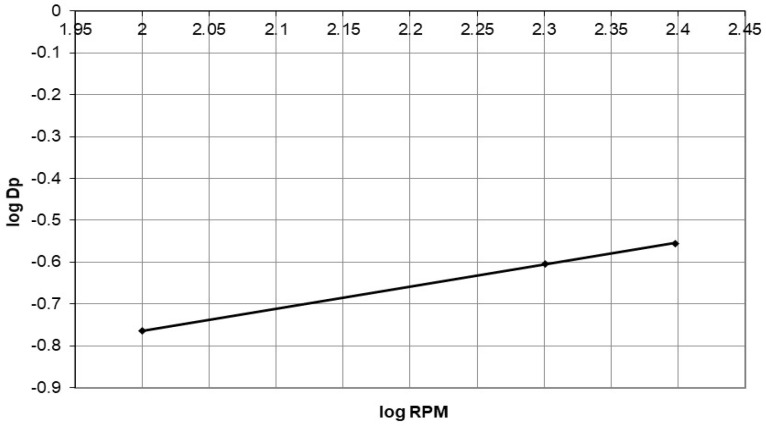
The plot of log D_p_ against log agitation speed for adsorption of cobalt ions on nanocellulose at different agitation speeds.

The D_p_ values, which are rate parameters, are determined from the linear region in Figure 17 and presented in Table 4. These values are also plotted in Figure 19 as a log D_p_ vs. log rpm graph. An equation of the general form [24] can be used to correlate the D_p_ values:

D_p_ = A (variable)^B^ (16)

In logarithmic form

Log D_p_ = log A + B log (variable) (17)

The data in Table 4 show the correlation between variables (agitation speed or initial cobalt concentration) and the correlation coefficient in Equation (17).

In Figure 18, the graph of q_t_/q_e_ against t^0.5^ is displayed for various initial concentrations. The linear portion of Figure 18 is used to calculate the rate parameter, D_p_, which is displayed in Table 4. The D_p_ values can also be correlated using Equation (17).

The initial cobalt concentration is variable, and the corresponding data can be found in Table 4 together with the correlation coefficient from Equation (17).

**Figure 20:**
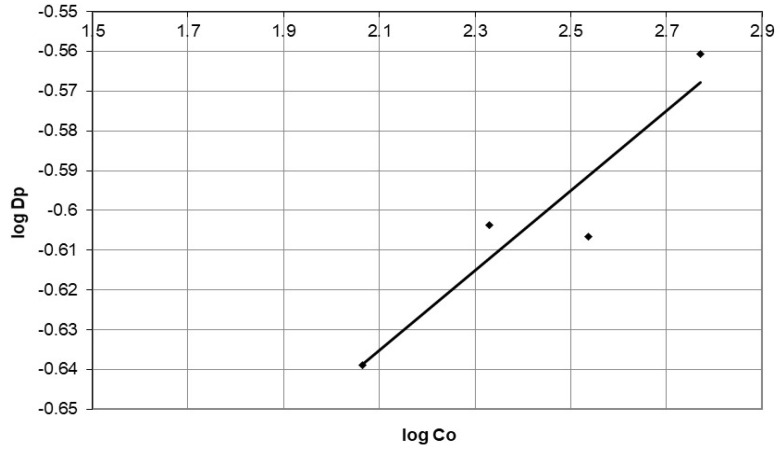
The plot of log D_p_ against log initial concentration for adsorption of cobalt ions on nanocellulose at different initial concentrations.

The plot of log D_p_ versus log C_o_ in Figure 20 shows that log D_p_ increases with increasing log C_o_, possibly due to a greater driving force for cobalt to penetrate solid particles. The plot has a slope of 0.1, which suggests that the adsorption mechanism of cobalt on nanocellulose is complex, involving both boundary-layer and intraparticle diffusion, as no exponential dependence on concentration 0.5 was observed [22]. The linearity of the plot suggests that diffusion within the particle is the most important rate-determining step, though there is also boundary-layer resistance. This behavior has been observed by McKay [22].

##### Comparison between the Kinetic Models

In this study, the adsorption of cobalt ions onto nanocellulose was investigated using reaction and diffusion models to identify the rate-limiting step, as revealed by the above results. The study considered two controlling situations: chemical-reaction controlling and cobalt-transport controlling. The optimal kinetic model and rate-limiting step were determined by analyzing the adsorption of cobalt ions on nanocellulose using the reaction and diffusion models. Various reaction models were calculated, including pseudo-first-order, pseudo-second-order, and Elovich, as well as the intraparticle diffusion model. The adsorption rate was affected by factors such as agitation speed and initial concentration of the cobalt solution.

The kinetic adsorption of cobalt ions on nanocellulose was analyzed using various models, namely the pseudo-first-order, pseudo-second-order, and Elovich models to investigate the rate of the chemical reaction between cobalt ions and nanocellulose as the primary controlling step. The results showed that the pseudo-first-order model had a relatively low correlation coefficient (R^2^) compared to the experimental values. The R^2^ is a statistical measure that indicates the linear relationship between two variables. A low correlation coefficient implies that the experimental data does not fit the model well, and other unaccounted factors might affect the adsorption process.

The R^2^ values obtained using the pseudo-first-order model ranged from 0.747 to 0.877, indicating a low to moderate correlation between the model and experimental data. However, this model was only accurate for the first 15 min of the experiment, and other reactions or processes might occur beyond that time frame. Therefore, the pseudo-first-order model may only be suitable for a short period.

In contrast, the second-order equation accurately represented the experimental data except for one instance, where the correlation coefficient was 0.577 for a limited time range of 0–3 min at an agitation speed of 100 rpm. This suggests that a chemical process might be the rate-determining factor. Additionally, the pseudo-second-order adsorption-rate constant (k_2_) of cobalt ions was controlled by a chemosorption interaction, supported by the significant value of k_2_, indicating a quicker sorption rate.

In summary, the study highlights the importance of using appropriate models to analyze the kinetic adsorption of cobalt ions on nanocellulose. While the pseudo-first-order model had limitations, the pseudo-second-order model provided more accurate results and insights into the adsorption process. These findings are consistent with previous studies on other ions, which have also demonstrated the dominance of the pseudo-second-order model in the adsorption processes involving valency forces through sharing or exchanging electrons between the solvent and the adsorbate [11,12,13,15,16,17]. In their work, Gomaa et al. [11] studied methylene blue (MB) adsorption on a hybrid mesoporous copper(II) oxide@barley straw-derived SiO_2_ (CuO@BSS) nanocomposite. They found that the pseudo-second-order kinetic model correctly described the kinetic behavior of MB adsorption on CuO@BSS nanocomposite. However, during the photocatalytic degradation of MB dye, the reaction mechanism fits better to the pseudo-first-order reaction kinetics, indicating that the MB-removal mechanism is physisorption during the photocatalytic degradation process using CuO@BSS nanocomposite under UV irradiation. Also, Gomaa et al. [12] used the pseudo-first/second-order kinetic models to evaluate the Fe^3+^ adsorption kinetics behavior using a hybrid spongy-like porous carbon-based on-the-azo pyrazole-benzenesulfonamide derivative. The findings show that the maximum saturation capacity is close to the experimental data according to the q_e_ of the pseudo-second-order model. On the other hand, the degradation of crystal violet through zinc oxide/silica fume-derived SiO_2_ nanocomposite was investigated by Kassem et al. [14]. Their findings showed that the R^2^ value of the pseudo-first-order degradation kinetic model was comparatively higher than the pseudo-second-order model. This suggests that the pseudo-first-order model can aptly describe the photodegradation of crystal violet by zinc oxide/silica fume-derived SiO_2_ nanocomposite.

The Elovich equation was used to model the results of cobalt ion adsorption on nano-cellulose at different initial concentrations and agitation speeds. The parameters obtained from this model showed a good fit to the experimental data, with a minimum correlation coefficient of 0.849. The high R^2^ values further confirm the success of the Elovich paradigm in describing cobalt-ion adsorption on nanocellulose, which may indicate a chemisorption process. The greater (α) values observed may be attributed to the higher surface area of the nanocellulose. The value of β decreased from 0.9141 g/mg to 0.7928 g/mg at a constant concentration and increasing agitation speed due to the increased surface coverage and some active sites, which is consistent with the findings of Gomaa et al. [12] on the highly selective Fe^3+^ adsorption from natural water samples using a hybrid spongy-like porous carbon-based on-azo-pyrazole-benzenesulfonamide derivative.

Understanding the efficiency of adsorbents in removing pollutants from wastewater requires a thorough understanding of the kinetics of adsorption. While pseudo-first-order and pseudo-second-order models have been employed to investigate adsorption, the intraparticle diffusion model is also necessary to clarify the diffusion mechanism. Therefore, the intraparticle diffusion model was used to clarify the controlling step of the adsorption rate, with intraparticle diffusion being the rate-limiting step. This model reveals that the adsorption process involves two key steps: the diffusion of cobalt ions into the interior pores of the nanocellulose and the migration of cobalt ions from the aqueous solution to the surface of the nanocellulose. The rate-limiting step is controlled by intraparticle diffusion when the plot passes through the origin. At the same time, if it does not, the adsorption mechanism is controlled by intraparticle diffusion and film diffusion. 

Table 4 lists the values for the macropore rate parameter (D_p_) and R^2^, which were obtained by calculating D_p_ from the slope of the line generated by plotting q_t_/q_m_ against t^0.5^. Figure 17 and Figure 18 demonstrate the dual nature of the curves, which is attributed to the varying extent of sorption in the initial and final stages of the experiment. The first, sharper portion corresponds to external-surface adsorption, whereas the second portion reflects gradual adsorption, where intraparticle diffusion is the rate-limiting step. If the lines do not pass through the origin point, this indicates the influence of film diffusion on adsorbate adsorption. External mass-transfer resistance has minimal impact on the adsorption rate. In contrast, internal diffusion resistance is the primary factor controlling the adsorption rate, particularly in the adsorption process after the initial fast-adsorption period. Similar trends were obtained by Gomaa et al. [12] and Ibrahim et al. [17]. 

Gomaa et al. [12] used the intraparticle diffusion model to clarify the controlling step of the adsorption rate, with intraparticle diffusion being the rate-limiting step to evaluate the Fe^3+^ adsorption-kinetics behavior using a hybrid spongy-like porous carbon-based on-the-azo pyrazole-benzenesulfonamide derivative. Ibrahim et al. [17] also employed Boyd film diffusion and intraparticle diffusion plots to investigate the rate-limiting step. Their findings indicate that the adsorption process comprises two critical steps for the intraparticle diffusion model: diffusion of the adsorbate within the interior pores of the adsorbent and migration of the solute from the solution into the outer surface of the adsorbent. They concluded that the adsorption mechanism is not solely governed by intraparticle diffusion since film diffusion also plays a role. However, when the plot passes through the origin, the rate-limiting step is controlled by intraparticle diffusion. 

The results of the study revealed that the pseudo-second-order and intraparticle models were the best fit for the experimental data. These models suggested that the mechanism of cobalt-ion removal involves general chemical reactions and diffusion resistance, indicating that the adsorption process is controlled by chemisorption. The Elovich model also provided a good fit for the experimental data and may indicate a chemisorption process. The kinetic results were subjected to further analysis by utilizing the intraparticle diffusion model to elucidate the diffusion mechanism. It was determined that the primary factor governing the adsorption rate is the internal diffusion resistance, with film diffusion playing a minor role.

The R^2^ value was used to assess the goodness of fit and identify-rate control mechanisms. However, no conclusive statement could be made about the rate-determining step based solely on the R^2^ results. Nonetheless, these results suggested that both mechanisms (chemical-reaction mechanism and mass-transfer resistance mechanism) may occur simultaneously in the adsorber. To distinguish between simultaneous reactions and internal mass-transfer resistance, the mass-transfer equation should include the reaction term. However, this critical analysis was beyond the study’s scope.

## 6. Comparison of Waste Palm Leaves-Derived Nanocellulose as an Adsorbent with Literature-Reported Adsorbents

The extraction of nanocellulose from waste palm leaves has several unique and significant advantages compared to the extraction from wastepaper and bagasse. 

First, palm leaves are abundant and widely available in many tropical regions, making them a low-cost and sustainable source of nanocellulose. In contrast, wastepaper and bagasse may not be as readily available in some regions or may be more expensive to collect and process. Secondly, palm leaves contain a higher percentage of cellulose compared to wastepaper and bagasse, which means that the extraction process can yield a greater amount of nanocellulose per unit of starting material. This makes palm leaves a more efficient source of nanocellulose. Thirdly, the properties of nanocellulose extracted from palm leaves may be different from those extracted from other sources, due to variations in the structure and composition of the starting material. This could lead to unique applications for nanocellulose extracted from palm leaves, such as in the development of biomaterials or composites.

Overall, the extraction of nanocellulose from waste palm leaves offers a promising avenue for the production of sustainable and high-value materials.

Table 5 shows that nanocellulose extracted from agricultural waste such as palm leaves is more effective in removing cobalt ions from wastewater than activated carbon extracted from hazelnut peel and orange peel waste indicates that studies have shown that activated carbon has a lower adsorption capacity and a slower adsorption rate compared to nanocellulose. 

It has been found that the chemical modification of chitosan enhances its ability to adsorb cobalt ions in wastewater. Modified chitosan has a similar adsorption capacity to nanocellulose. Activated disordered mesoporous carbon has a disordered porous structure and high surface area but is not as effective as nanocellulose in removing cobalt ions from wastewater. The capacity of cobalt ion adsorption is inferior to that of nanocellulose even though sediments from a dam can be utilized as adsorbents for eliminating heavy metals from wastewater.

Choosing the right sorbent depends on many factors, including concentrations, costs, availability, and environmental impact of heavy metals. Surface chemistry, particle size, and morphology are also important factors affecting adsorption performance.

The performance of nanocellulose prepared from waste palm leaves was comparable to those of the adsorbents reported in the literature [28,39,40,41,42], as shown in Table 5. The production of nanocellulose is very promising, the cost of waste palm leaves is low, the material is obtained locally, and it is a very cheap material. Other sorbents in Table 5 were also made from natural materials. Based on this result, the nanocellulose produced in this work showed competitive performance compared to the sorbents in Table 5. Overall, the advantages of using nanocellulose as an adsorbent are low cost and ease of preparation. The disadvantages are that its maximum adsorption capacity may not be as high as other commercial adsorbents, and the synthesis of some commercial adsorbents can be complex and expensive.

## 7. Assessing the Stability of Nanocellulose Composite for Cobalt Ion Removal

Ensuring the stability of an adsorbent material is a crucial step in assessing its effectiveness in adsorption processes. One method of verifying stability is examining experimental evidence, such as Fourier transform infrared (FTIR) analysis. In this study, FTIR tests were conducted both before and after the adsorption process to assess any changes in the chemical composition of a nanocellulose composite.

After adsorption, the nanocellulose was separated from the solution mixture through filtration, washed with distilled water, and dried at 60 °C for 8 h. The dried nanocellulose was then treated with a 0.1 M hydrochloric acid solution at room temperature for 12 h to remove the cobalt ions. Finally, the regenerated material was filtered, washed, and dried for further adsorption experiments. This process was repeated for three consecutive cycles.

Figure 5 shows that the FT-IR spectrum analysis of nanocellulose before and after the adsorption process, following three consecutive adsorption–desorption cycles, did not indicate any significant changes in the nanocellulose composite’s composition. These results suggest that the composite remained stable even after undergoing the adsorption process and subsequent regeneration cycles and was not susceptible to degradation or other unwanted effects. Furthermore, the adsorption capacity (5.98 mg/g) remained consistent after each cycle, indicating that the regenerated material could still effectively remove cobalt ions.

Nanocellulose is an intriguing material that exhibits excellent potential for environmental and health applications, particularly in the remediation of toxic metals from water bodies. Furthermore, its exceptional physical properties, such as high biodegradability, biocompatibility, and low cytotoxicity, have garnered significant attention in various fields of application. Unlike inorganic nanoadsorbents, cellulose nanofibrils possess low genotoxicity and high biodegradability [43]. As a result, nanocellulose is widely considered a safe and biocompatible material that poses minimal risk to human health and the environment. 

The use of nanocellulose composites for the adsorption of cobalt ions from wastewater is considered safe for human consumption, thanks to the material’s biocompatibility, biodegradability, and nontoxic nature. However, ensuring proper synthesis, processing, and purification of the composite is essential to prevent water contamination. If contamination is detected, physical methods such as filtration, centrifugation, or sedimentation can separate the composite particles from the water.

Moreover, chemical treatments such as coagulation flocculation can enhance the removal efficiency of the composite further. Therefore, it is crucial to employ appropriate purification methods to guarantee the safety of the water for human consumption. With these precautions in place, using nanocellulose composites to remove toxic metals from water bodies holds great promise for ensuring a healthier and safer environment.

## 8. The Efficiency of Nanocellulose as an Adsorbent for Real Industrial Wastewater Treatment: A Study on Heavy Metal Removal

The purpose of this study was to assess the effectiveness of nanocellulose as an adsorbent in the treatment of real industrial wastewater, specifically focusing on the removal of heavy metals. The industrial wastewater used in the study was obtained from the industrial area in Riyadh and was analyzed using the PerkinElmer AAnalyst 700 atomic spectrometer, with the results presented in Table 6. The heavy metals found in the industrial wastewater originated from various factories involved in metal plating, ceramic painting, paint production, catalysts manufacturing, alloy production, wire conducting, galvanizing iron, polymer stabilizer production, battery manufacturing, semiconductor production, pesticide production, wood preservation, and pigment production.

According to Table 6, heavy metal concentration levels in industrial wastewater were above permissible levels, except for lead and zinc ions, compared to levels required for crop production as noted by Pescode [44]. Nanocellulose was utilized as an adsorbent and removed heavy metals from real wastewater with removal efficiency ranging from 97.52% to 100%. The study demonstrated that nanocellulose could effectively eliminate mixed industrial wastewater, reducing heavy metal concentration levels below permissible levels for crop production according to Pescode [44]. This study highlights the practical applicability and efficiency of nanocellulose as an adsorbent for real industrial wastewater treatment.

## 9. Conclusions

In this study, waste palm-leaf-derived nanocellulose has been found to be a promising and efficient adsorbent for removing cobalt ions from industrial wastewater. The nanocellulose has an average size of 85 nm and an average pore width of 11.13 nm, with a maximum cobalt-ion adsorption capacity at room temperature of 5.98 mg/g. The experimental results were consistent with the Langmuir adsorption model, which assumes metal ions are adsorbed as a monolayer. The study tested agitation speeds of 100 and 200, and 250 rpm and found that higher speeds increase the rate of cobalt ion adsorption on nanocellulose. At low speeds, the mass transfer becomes the limiting step. When initial cobalt ion concentrations varied from 100 to 600 ppm at a constant 200 rpm, lower concentrations decreased faster due to limited available binding sites on the nanocellulose surface.

The study employed various kinetic models, and the pseudo-second-order model provided more accurate insights into the chemisorption-controlled adsorption process. FT-IR spectrum analysis showed that the nanocellulose composite remained stable even after undergoing the adsorption process and subsequent regeneration cycles, making it a practical and low-cost option for industrial applications. The study demonstrated the effectiveness of nanocellulose in removing heavy metals from real industrial wastewater, with a removal efficiency between 97.52% and 100%, below permissible levels for crop production. This highlights nanocellulose’s potential as an ecofriendly solution for heavy metal removal from industrial wastewater. Overall, this study provides valuable insights into the efficacy of nanocellulose as an adsorbent for heavy metal removal from wastewater, with potential for further research in other industrial applications.

## Figures and Tables

**Table 1:** The average pore size.

Element	Nanocellulose
Average pore width (4 V/A by BET), nm	11.13

**Table 2:** Isotherm Constants for cobalt ions adsorption on nanocellulose.

Langmuir Isotherm			Freundlich Isotherm		
K (L/g)	b (L/mg)	R^2^	kF (L/g)	*n* (-)	R^2^
0.0801	0.0108	0.97	0.282	1.8	0.85

**Table 3:** Kinetic Models Parameters for cobalt ions adsorption on nanocellulose.

Adsorbent Parameters	Pseudo-First-Order		Pseudo-Second-Order		Elovich		
	k_1_ (min^−1^)	R^2^	k_2_ (g/mg·min)	R^2^	œ (mg/g·min)	ß (g/mg)	R^2^
Initial conc. (mg/L)							
116	0.1873	0.898	0.0556	0.991	3.275	0.5925	0.970
214	0.1357	0.763	0.0520	0.991	3.053	0.7963	0.913
344.8	0.1427	0.757	0.0096	0.934	0.659	1.228	0.924
589	0.0972	0.748	0.0134	0.931	0.906	1.233	0.851
Agitation speed (rpm)							
100	0.1376	0.747	0.0044	0.577	0.663	0.9141	0.946
200	0.1357	0.763	0.0520	0.991	3.053	0.7963	0.913
250	0.1446	0.877	0.0865	0.997	6.004	0.7928	0.849

**Table 4:** Intraparticle diffusion parameter for cobalt ions adsorption on nanocellulose.

Initial Concentration (mg/L)		D_p_ (mg/g·min 0.5)	R^2^
116		0.2297	0.946
214		0.249	0.966
344.8		0.247	0.999
589		0.275	0.763
A	0.143		
B	0.101		
R^2^	0.86		
Agitation speed (rpm)			
100		0.172	0.955
200		0.249	0.966
250		0.278	0.931
A	0.0153		
B	0.5263		
R^2^	0.99		

**Table 5:** A comparison of prepared nanocellulose with other reported literature results.

Ref	Adsorbent Type	Solution	T (°C)	Capacity (mg/g)
[28]	Activated Carbon Prepared from Hazelnut Shells	Cobalt ions	25	3.8
[39]	Orange Peel Waste	Cobalt ions	25	4.25
[40]	Chemically Modified Chitosan	Cobalt ions	25	5.89
[41]	Activated Disordered Mesoporous Carbons	Cobalt ions	25	2
[42]	Sediments From a Dam	Cobalt ions	25	0.93
This work	Nanocellulose	Cobalt ions	25	5.98

**Table 6:** Real industrial wastewater.

	Adsorption by Nanocellulose		
Heavy Metals	Concentration before Treatment (In the Collected Real Wastewater)	Concentration after Treatment	Treatment Efficiency %
Co	3.97	0.003	99.92
Cu	3.58	0.007	99.80
Zn	0.83	0	100
Pb	2.71	0.002	99.93
As	3.14	0.078	97.52
Cd	2.87	0.001	99.97
Cr	1.85	0.001	99.95

## Data Availability

Not applicable.

## References

[1] Wang R., Deng L., Fan X., Li K., Lu H., Li W. (2021). Removal of heavy metal ion cobalt (II) from wastewater via adsorption method using microcrystalline cellulose–magnesium hydroxide. Int. J. Biol. Macromol..

[2] Varghese A.G., Paul S.A., Latha M.S. (2018). Remediation of heavy metals and dyes from wastewater using cellulose-based adsorbents. Environ. Chem. Lett..

[3] Zhang W., Zhao J., Ao C., Lu C. (2021). Nanocellulose Materials in Water Treatment. Video Proc. Adv. Mater..

[4] Khalilzadeh M.A., Hosseini S.E., Rad A.S., Venditti R.A. (2020). Synthesis of grafted nano-fibrillated cellulose-based hydrogel and study of its thermodynamic, kinetic, and electronic properties. J. Agric. Food Chem..

[5] Ogwang G., Olupot P., Kasedde H., Menya E., Storz H., Kiros Y. (2021). Experimental evaluation of rice husk ash for applications in geopolymer mortars. J. Bioresour. Bioprod..

[6] Obey G., Adelaide M., Ramaraj R. (2022). Biochar derived from non-customized matamba fruit shell as an adsorbent for wastewater treatment. J. Bioresour. Bioprod..

[7] Hasan I., El-Din S., AbdElRaady A. (2022). Peppermint-Mediated Green Synthesis of Nano ZrO_2_ and Its Adsorptive Removal of Cobalt from Water. Inorganics.

[8] Gad H., Omar H., Aziz M., Hassan M., Khalil M. (2016). Treatment of Rice Husk Ash to Improve Adsorption Capacity of Cobalt from Aqueous Solution. Asian J. Chem..

[9] Swelam A., Salem A., Ayman A., Farghly A. (2018). Kinetic and Thermodynamic Sorption Study of Cobalt Removal from Water Solution with Magnetic Nano-Hydroxyapatite. Al Azhar Bull. Sci..

[10] Essaadaoui Y., Lebkiri A., Rifi E., Kadiri L., Ouass A. (2018). Adsorption of cobalt from aqueous solutions onto Bark of Eucalyptus. Mediterr. J. Chem..

[11] Gomaa H., Hussein M.A., Motawea M.M., Aboraia A.M., Cheira M.F., Alotaibi M.T., El-Bahy S.M., Ali H.M. (2022). A hybrid mesoporous CuO@barley straw-derived SiO_2_ nanocomposite for adsorption and photocatalytic degradation of methylene blue from real wastewater. Colloids Surf. A Physicochem. Eng. Asp..

[12] Gomaa H., Sayed A., Mahross M., Mohamed A., Ismail M., Othman A., Jiansheng B., El-Bahy M. (2022). A hybrid spongy-like porous carbon-based on azopyrazole-benzenesulfonamide derivative for highly selective Fe^3+^-adsorption from real water samples. Microporous Mesoporous Mater..

[13] Gomaa H., El-Monaem E., Eltaweil A., Omer A. (2022). Efcient removal of noxious methylene blue and crystal violet dyes at neutral conditions by reusable montmorillonite/NiFe_2_O_4_@amine-functionalized chitosan composite. Sci. Rep..

[14] Kassem K., Hussein M., Motawea M., Alrowaili Z., Ezzeldien M. (2021). Design of mesoporous ZnO @ silica fume-derived SiO_2_ nanocomposite as photocatalyst for efficient crystal violet removal: Effective route to recycle industrial waste. J. Clean. Prod..

[15] Salama R., El-Hakam S., Samra S., El-Dafrawy S., Ibrahim A., Ahmed A. (2022). Synthesis, characterization of titania supported on mesoporous MCM-41 and its application for the removal of methylene blue. Delta Univ. Sci. J..

[16] Bakry A., Alamier W., Salama R., El-Shall M., Awad F. (2022). Remediation of water containing phosphate using ceria nanoparticles decorated partially reduced graphene oxide (CeO_2_-PRGO) composite. Surf. Interfaces.

[17] Ibrahim A., Salama R., El-Hakam S., Khder A., Ahmed A. (2021). Synthesis of 12-tungestophosphoric acid supported on Zr/MCM-41 composite with excellent heterogeneous catalyst and promising adsorbent of methylene blue. Colloids Surf. A Physicochem. Eng. Asp..

[18] Kyzas G.Z., Favvas E.P., Kostoglou M., Mitropoulos A.C. (2020). Effect of agitation on batch adsorption process facilitated by using nanobubbles. Colloids Surf. A Physicochem. Eng. Asp..

[19] Quiton K.G.N., Huang Y.H., Lu M.C. (2022). Recovery of cobalt and copper from single and co-contaminated simulated electroplating wastewater via carbonate and hydroxide precipitation. Sustain. Environ. Res..

[20] Sparks L. (2018). Soil Physical Chemistry.

[21] Do D. (1998). Adsorption Analysis: Equilibria and Kinetics.

[22] Mckay G. (1983). The adsorption of dyestuffs from aqueous solutions using activated carbon. III. Intraparticle diffusion processes. J. Chem. Technol. Biotechnol..

[23] Mckay G., Otterbun S., Sweeney G. (1980). The removal of color from effluents using various adsorbents-III. Silica: Rate process. Water Res..

[24] Crank J. (1965). The Mathematics of Diffusion.

[25] Nazir M., Wahjoedi B., Yussof A., Abdullah M. (2013). Eco-friendly extraction and characterization of cellulose from oil palm empty fruit bunches. BioResources.

[26] EL-Geundi M. (1990). Adsorption Equilibria of Basic Dyestuffs onto Maize Cob. Adsorp. Sci. Technol..

[27] Suresh S., Srivastava V., Mishra I. (2011). Study of Catechol and Resorcinol Adsorption Mechanism through Granular Activated Carbon Characterization, pH and Kinetic Study. Sep. Sci. Technol. Sep. Sci. Technol..

[28] Demirbaş E. (2003). Adsorption of cobalt (II) ions from aqueous solution onto activated carbon prepared from hazelnut shells. Adsorpt. Sci. Technol..

[29] Qasem A., Mohammed H., Lawal U. (2021). Removal of heavy metal ions from wastewater: A comprehensive and critical review. npj Clean Water.

[30] Samyuktha S., Shreya k., Sadamanti A., Sreedhar I., Kale S. (2021). Heavy metal removal from wastewater using nanomaterials, process and engineering aspects. Process Saf. Environ. Prot..

[31] Mckay G., Ho S. (2002). Application of kinetic models to the sorption of copper (II) on to peat. Adsorpt. Sci. Technol..

[32] Averettet C., Leenheer A., Mcknight M., Thorn A. (1994). Humic Substances in the Suwanne River, Georgia: Interactions, Properties, Proposed Structure.

[33] Leenheer A., Brown K., McCarthy P., Cabaniss E. (1998). Models of Metal Binding Structures in Fulvic Acid from the Suwannee River, Georgia. Environ. Sci. Technol..

[34] Juang S., Tseng L., Wu C., Lee H. (1997). Adsorption behavior of reactive dyes from aquous solutions on chitosan. J. Chem. Technol. Biotechnol..

[35] Yadava P., Tyagi S., Singh N. (1991). Effect of temperature on the removal of lead(II) by adsorption on China clay and wollastonite. J. Chem. Technol. Biotechnol..

[36] Juang S., Wu C., Lee H. (1996). Liquid-phase adsorption of phenol and its derivatives on activated carbon fibers. Sep. Sci. Technol..

[37] Juang S., Swei L. (1996). Effect of dye nature on its adsorption from aqueous solutions onto activated carbon. Sep. Sci. Technol..

[38] Stuart X., Camp T. (1973). Solution of the fixed bed physical adsorption problem with two significant rate controlling steps. AIChE Symp. Ser..

[39] Yalçın Altunkaynak Y., Canpolat M., Yavuz Ö. (2022). Adsorption of cobalt (II) ions from aqueous solution using orange peel waste: Equilibrium, kinetic and thermodynamic studies. J. Iran. Chem. Soc..

[40] Al-Shahrani A., Fadi Alakhras F., Al-Mazaideh G. (2018). Sorption of Cobalt (II) Ions from Aqueous Solutions Using Chemically Modified Chitosan. Glob. NEST J..

[41] Bernabé I., Gómez J., Díez E., Sáez P., Rodríguez A. (2019). Optimization and Adsorption-Based Recovery of Cobalt Using Activated Disordered Mesoporous Carbons. Adv. Mater. Sci. Eng..

[42] Oca-Palma R., Solache-Ríos M., Jiménez-Reye M., García-Sánchez J., Almazán-Sánchez P. (2021). Adsorption of cobalt by using inorganic components of sediment samples from water bodies. Int. J. Sediment Res..

[43] Ramos-Vargas S., Huirache-Acuña R., Rutiaga-Quiñones J., Cortés-Martínez R. (2020). Effective lead removal from aqueous solutions using cellulose nanofibers obtained from water hyacinth. Water Supply.

[44] Pescode M.B. (1992). Wastewater Treatment and Use in Agriculture.

